# Cost-Effectiveness of Adjuvant Olaparib for Patients With Breast Cancer and Germline *BRCA1/2* Mutations

**DOI:** 10.1001/jamanetworkopen.2023.50067

**Published:** 2024-01-03

**Authors:** Christina M. Zettler, Dilanka L. De Silva, Victoria S. Blinder, Mark E. Robson, Elena B. Elkin

**Affiliations:** 1 COTA Healthcare, New York, New York; 2 Peter MacCallum Cancer Centre, Parkville Familial Cancer Centre, Melbourne, Victoria, Australia; 3 Thoracic Oncology Service, Memorial Sloan Kettering Cancer Center, New York, New York; 4 Breast Medicine Service and Immigrant Health and Cancer Disparities Service, Memorial Sloan Kettering Cancer Center, New York, New York; 5 Breast Medicine Service, Memorial Sloan Kettering Cancer Center, New York, New York; 6 Department of Health Policy and Management, Columbia University Mailman School of Public Health, New York, New York

## Abstract

**Importance:**

The OlympiA trial found that 1 year of adjuvant olaparib therapy can improve distant disease-free survival and overall survival from early-stage breast cancer in patients with a germline *BRCA1/2* mutation. However, olaparib, an oral poly-adenosine diphosphate ribose polymerase inhibitor, is estimated to cost approximately $14 000 per month in the US.

**Objective:**

To estimate the incremental cost-effectiveness of adjuvant olaparib compared with no olaparib in eligible patients.

**Design, Setting, and Participants:**

In an economic evaluation from a health care system perspective, the cost-effectiveness of adjuvant olaparib was analyzed using a Markov state-transition model. The model simulated costs and lifetime health outcomes of 42-year-old women with high-risk early-stage breast cancer and a known *BRCA1/2* mutation who completed definitive primary therapy and neoadjuvant or adjuvant systemic therapy. The study was conducted from August 2021 to July 2023. The effectiveness of olaparib was based on the findings of the OlympiA randomized clinical trial, and other model parameters were identified from the literature. The model was calibrated to the 1-, 2-, 3-, and 4-year distant disease-free and overall survival observed in the OlympiA trial, and olaparib was assumed to reduce the risk of distant recurrence only in the first 4 years.

**Exposure:**

One year of adjuvant olaparib or no adjuvant olaparib.

**Main Outcome and Measure:**

Incremental cost-effectiveness ratio (ICER) in 2021 US dollars per quality-adjusted life-year (QALY) gained. All outcomes were discounted by 3% annually.

**Results:**

In the base case, adjuvant olaparib was associated with a 1.25-year increase in life expectancy and a 1.20-QALY increase at an incremental cost of $133 133 compared with no olaparib. The resulting ICER was approximately $111 000 per QALY gained. At a willingness-to-pay threshold of $150 000 per QALY, olaparib was cost-effective at its 2021 price and in more than 92% of simulations in probabilistic sensitivity analysis. The results were sensitive to assumptions about the effectiveness of olaparib and quality of life for patients with no disease recurrence.

**Conclusions and Relevance:**

In this study, from a US health care system perspective, adjuvant olaparib was a cost-effective option for patients with high-risk, early-stage breast cancer and a germline *BRCA1/2* mutation.

## Key Points

**Question:**

Is adjuvant olaparib cost-effective in patients with early-stage breast cancer with high-risk disease and germline *BRCA1/2* mutations?

**Findings:**

In an economic evaluation using a Markov model, adjuvant olaparib therapy was associated with a 1.25-year increase in life expectancy and a 1.20-quality-adjusted life-year (QALY) increase at an incremental cost of $133 133 compared with no olaparib, yielding a discounted incremental cost-effectiveness ratio of approximately $111 000 per QALY gained. Results were sensitive to assumptions about the effectiveness of olaparib and quality of life for patients with no disease recurrence.

**Meaning:**

The findings of this study suggest that, at a willingness-to-pay threshold of $150 000 per QALY, adjuvant olaparib may be cost-effective at its 2021 price.

## Teaser

This economic evaluation examines the cost-effectiveness of adjuvant olaparib therapy in individuals with early-stage breast cancer and a germline *BRCA1/2* mutation.

## Introduction

Olaparib, a poly-adenosine diphosphate ribose polymerase (PARP) inhibitor, was first approved by the US Food and Drug Administration in 2014 for the treatment of advanced ovarian cancer in patients with a deleterious or suspected deleterious germline *BRCA1/2* mutation (gBRCAm). In 2018, it was approved in the US for *ERBB2* (previously *HER-2*)-negative metastatic breast cancer associated with a gBRCAm in patients who previously received chemotherapy.^1^

In June 2021, interim results of the randomized, double-blind OlympiA clinical trial were published, demonstrating the efficacy of olaparib for treating breast cancer in the adjuvant setting.^2^ Patients with pathogenic or likely pathogenic gBRCAm and nonmetastatic, *ERBB2*-negative primary breast cancer were randomized to receive twice-daily oral olaparib or placebo for 1 year following completion of definitive local treatment and neoadjuvant or adjuvant chemotherapy. In March 2022, an updated interim analysis^3^ with 1836 patients, 330 invasive-disease-free survival events, and a median of 3.5 years of follow-up reported that patients receiving olaparib had superior 4-year distant disease-free survival (DDFS) (86.5% vs 79.1% for placebo) and overall survival (OS) (89.8% vs 86.4% for placebo). The hazard ratio (HR) for distant disease or death at 4 years was 0.61 (95% CI, 0.48-0.77). Olaparib was subsequently approved for use in the adjuvant setting among patients similar to those in the OlympiA trial.

While approximately 3% of all breast cancers in the US are associated with a gBRCAm, pathogenic variants of these genes are prevalent in more than 10% of patients with triple-negative breast cancer (TNBC)—tumors that lack estrogen and progesterone receptors and do not overexpress ERBB2 protein.^4,5,6^ Triple-negative breast cancer generally has more limited therapeutic options and poorer prognosis than other types of breast cancer.^7,8^ In the OlympiA trial, more than 80% of patients in both arms had TNBC.

The results of the OlympiA trial indicate that adjuvant olaparib provides a clinically and statistically significant health benefit for patients with early-stage breast cancer and a known gBRCAm. Our goal was to assess the value of these improvements relative to olaparib’s cost.

## Methods

### Model

In an economic evaluation conducted from the health care system perspective, we created a Markov model to estimate the lifetime costs, life-years, and quality-adjusted life-years (QALYs) associated with adjuvant olaparib or no olaparib for early-stage breast cancer with known gBRCAm. The model included 4 health states: no recurrence, metastatic recurrence, death due to breast cancer, and death from other causes. All patients started in the no recurrence health state after completion of definitive primary therapy and neoadjuvant or adjuvant chemotherapy. From no recurrence, patients could transition to metastatic recurrence or die from other causes. Patients could only die from breast cancer in the metastatic recurrence health state. Local recurrences and other primary cancers were not modeled. The model used 1-month cycles and simulated the DDFS and OS observed in the OlympiA trial for the first 48 months. We extrapolated beyond this period by applying monthly estimates of metastatic recurrence from studies of patients with breast cancer with similar tumor features.^9,10^ The model was created in TreeAge Pro 2020 software (TreeAge Software LLC). The study was conducted from August 2021 to July 2023. The study was conducted using information from published reports in the peer-reviewed literature and did not involve new collection or analysis of data from humans; therefore, the study did not require institutional review board review or exemption according to the Common Rule (45 CFR §46). We followed the Consolidated Health Economic Evaluation Reporting Standards (CHEERS) reporting guideline.

### Clinical Data and Transition Probabilities

Consistent with the OlympiA trial,^2^ we assumed that patients received either oral olaparib, 300 mg, twice daily or no olaparib. The starting age was 42 years, which was the median age of patients in the olaparib arm of the trial. We also examined starting ages of 50 and 60 years.

The probability of metastatic recurrence without olaparib was derived from the DDFS results observed in the placebo arm of the OlympiA study. We estimated the annual rate of metastatic recurrence in each of the first 4 years and translated these year-specific rates into constant monthly probabilities.^11^ We modeled the association between olaparib and risk of metastatic recurrence in the first 4 years using the stratified HR for distant disease or death observed in the trial (HR, 0.61; 95% CI, 0.48-0.77). We assumed that olaparib did not reduce the risk of metastatic recurrence after 4 years. Consistent with the literature^9,10^ on recurrence risks for patients with TNBC, we assumed that the risk of metastatic recurrence decreased after the first 4 years and remained constant after 10 years.

Monthly rates of metastatic recurrence in the model were iteratively adjusted to recreate the 12-, 24-, 36-, and 48-month DDFS and OS observed in the OlympiA trial. We converted the reported annual recurrence probabilities to constant monthly rates within each year, then increased or decreased each monthly rate by increments of 0.0001 until the rates converted to monthly probabilities yielded the arm- and year-specific DDFS and OS estimates from the trial. The DDFS rates for the olaparib arm in the OlympiA trial were 94.4% at 12 months, 90.6% at 24 months, 88.0% at 36 months, and 86.5% at 48 months.^3^ For the no olaparib arm, the DDFS rates were 90.3% at 12 months, 84.0% at 24 months, 81.0% at 36 months, and 79.1% at 48 months. The OS based on this interim analysis for the olaparib arm was 98.0% at 12 months, 95.0% at 24 months, 92.8% at 36 months, and 89.8% at 48 months. For the no olaparib arm, the OS was 96.9% at 12 months, 92.8% at 24 months, 89.1% at 36 months, and 86.4% at 48 months.

Age-specific non–breast cancer mortality was estimated from 2018 US life tables.^12^ The monthly probability of death after metastatic recurrence was based on a published estimate of 13.3 months median survival in patients with TNBC.^13^

### Costs and Utilities

Using a health care system perspective, we estimated direct medical costs related to breast cancer, including olaparib, routine oncology visits, annual surveillance imaging, and the cost of care for metastatic recurrence. Olaparib was discontinued after the first of metastatic recurrence or 1 year of treatment. We assumed that patients receiving olaparib would see their oncologist monthly during the year of treatment, then every 6 months for 5 years after completing systemic therapy, and annually thereafter. Patients not receiving olaparib were assumed to have an oncologist visit every 6 months through year 5 and annually thereafter. We assumed that patients who had at least 1 breast (53.5% of patients in the OlympiA trial) would receive a diagnostic mammogram and breast magnetic resonance imaging with contrast annually. The costs of managing serious adverse events were not considered, given the tolerability profile of olaparib found in the OlympiA trial. Treatment costs for metastatic recurrence did not differ by receipt of adjuvant olaparib.

The costs of olaparib, routine oncology visits, breast imaging, and care for metastatic recurrence were identified from previously published studies, inflated to 2021 US dollars, and from the 2021 Medicare Fee Schedule (Table 1).^15,16,17,18^ Because most patients in the OlympiA trial had TNBC in both the olaparib (81.5%) and placebo (82.8%) arms, estimates for this specific patient population were used when available. All costs and health outcomes were discounted by 3% annually.^19^

**Table 1.** Model Inputs and Assumptions

Model input	Base-case value (range)	Distribution	Source
Utilities			
Annual utility for no recurrence/no adjuvant treatment^a^	0.98 (0.735-1)	Beta	Millar and Millward,^14^ 2007
Annual utility for metastatic recurrence	0.55 (0.413-0.688)	Beta	Millar and Millward,^14^ 2007
Utility multiplier for olaparib	0.9 (0.80-1)	Beta	Assumed
Costs (2021), $			
Monthly cost of olaparib	14 523 (10 892-18 154)	Gamma	Wu and Zhong,^15^ 2019
Cost of oncologist visit	867 (650-1084)	Gamma	Kurian et al,^16^ 2007
Cost of breast MRI and mammogram	540 (405-675)	Gamma	Medicare Physician Fee Schedule,^18^ 2021
Monthly cost of metastatic recurrence	23 599 (17 699-29 499)	Gamma	Skinner et al,^17^ 2021
Transition probabilities			
Hazard ratio for olaparib (metastatic recurrence)^b^	0.61 (0.48-0.77)	Log-normal	Tutt et al,^3^ 2022
Annual probability of metastatic recurrence, years 5-9	0.0156^c^	NV	Reddy et al,^9^ 2018; Steward et al,^10^ 2014
Annual probability of metastatic recurrence, years ≥10	0.0078^c^	NV	Reddy et al,^9^ 2018; Steward et al,^10^2014
Monthly rate of death following metastatic recurrence	0.052 (0.039-0.065)	Beta	Yin et al,^13^ 2020

Abbreviations: MRI, magnetic resonance imaging; NV, not varied.

^a^ Asymmetrical CI; SE was calculated based on the lower bound.

^b^ Range reflects the 95% CI from the OlympiA trial. For all other model inputs, range is based on values reported in the literature or ±25% of base-case value.

^c^ These estimates were not varied in sensitivity analysis.

The utility weight, which reflects health-related quality of life on a scale of 0 (worst imaginable health) to 1 (best imaginable health), for patients not receiving olaparib was assumed to be equivalent to the utility associated with no recurrence after completion of adjuvant treatment. This utility was 0.98, based on published estimates.^14^ The utility weight for patients receiving olaparib was estimated as a multiplicative function of the utility for patients not receiving olaparib and a factor less than 1. The utility for metastatic recurrence was estimated from published studies.^14^

### Sensitivity Analysis

We performed 1-way sensitivity analysis to evaluate the uncertainty around model inputs. Ranges of values were obtained from published studies or were varied ±25% from their base-case value (Table 1). We also performed probabilistic sensitivity analysis, assigning probability distributions to model inputs and conducting 10 000-second-order Monte Carlo simulations. Costs were characterized by γ distributions and utilities by β distributions. The HR for the association between olaparib and metastatic recurrence was modeled using a log-normal distribution, and the monthly rate of death following metastatic recurrence was modeled with a β distribution. Probabilistic sensitivity analysis results were plotted in a scatterplot of ICERs and a cost-effectiveness acceptability curve, varying the willingness-to-pay threshold from 0 to $300 000 per QALY.

## Results

### Base Case

In the base-case analysis, treatment with olaparib yielded 17.94 life-years and 17.40 QALYs at an average cost of $314 789 compared with no olaparib, which yielded 16.69 life-years and 16.20 QALYs at a cost of $181 655 (Table 2). Adjuvant olaparib was associated with a 1.25-year increase in life expectancy and a 1.20-QALY increase at an incremental cost of $133 133. The resulting ICERs were $106 506 per life-year and $110 962 per QALY gained. At a willingness-to-pay threshold of $150 000 per QALY gained, olaparib would be considered cost-effective at its 2021 price. Varying the age of the cohort resulted in a modest change in the results. Holding constant assumptions about disease recurrence and drug efficacy, adjuvant olaparib was associated with ICERs of $124 897 per QALY in patients aged 50 years and $154 921 per QALY in those aged 60 years.

**Table 2.** Base-Case Results^a^

Outcome	Olaparib	No olaparib	Incremental
Life-years	17.94	16.69	1.25
QALYs	17.40	16.20	1.20
Cost, $	314 789	181 655	133 133
$ per Life-year gained	NA	NA	106 506
$ per QALY gained	NA	NA	110 962

Abbreviations: NA, not applicable; QALYs, quality-adjusted life-years.

^a^ All outcomes (costs, life-years, and QALYs) were discounted by 3% annually.

### Sensitivity Analysis

**Figure 1.**
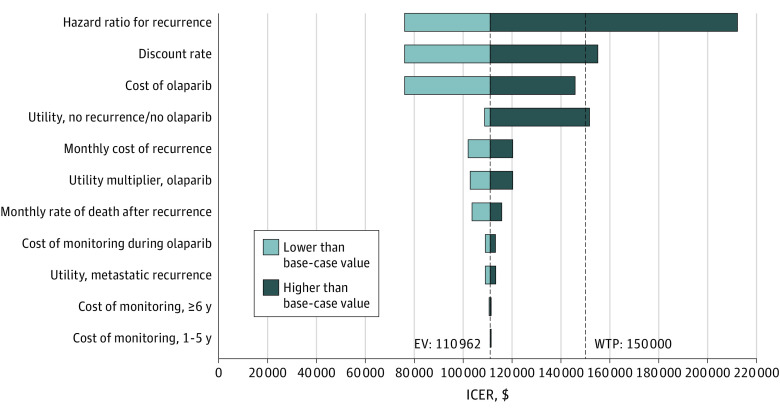
One-Way Sensitivity Analysis Tornado diagram shows results of one-way sensitivity analysis for key model inputs. The gray portion of each bar represents the incremental cost-effectiveness ratio (ICER) range when the specified model input is lower than its base-case value. The black portion of each bar represents the ICER range when the specified model input is higher than its base-case value. Low and high values for all parameters are presented in Table 1. The first vertical line reflects the base-case value of each input, where the ICER is $110 962 per quality-adjusted life-year (QALY) gained comparing adjuvant olaparib with no adjuvant olaparib. The second vertical line reflects the value of each input that would yield an ICER of $150 000 per QALY, holding all other inputs constant. Threshold values above or below which the ICER exceeded $150 000 per QALY were 0.694 for the hazard ratio for recurrence with olaparib; 0.048 for the discount rate; and 0.743 for the utility of no recurrence/no olaparib, holding all other parameters constant at their base-case values. EV indicates expected value; WTP, willingness to pay.

The ICER was most sensitive to assumptions about the effectiveness of olaparib, the discount rate, and the utility of the no recurrence health state (Figure 1). At the upper bound of the 95% CI for the HR for distant disease or death estimated in the OlympiA trial (0.77), adjuvant olaparib was associated with a cost of $212 303 per QALY gained. At the lower bound of the 95% CI (0.48), the ICER was $75 999 per QALY gained. When olaparib cost 25% more than the base case value ($18 154), the ICER was $145 868 per QALY gained. At the lower bound of the cost of olaparib ($10 892), the ICER was $76 056 per QALY gained. Results were least sensitive to the utility weight for metastatic recurrence and costs of routine oncology visits and surveillance mammograms.

**Figure 2.**
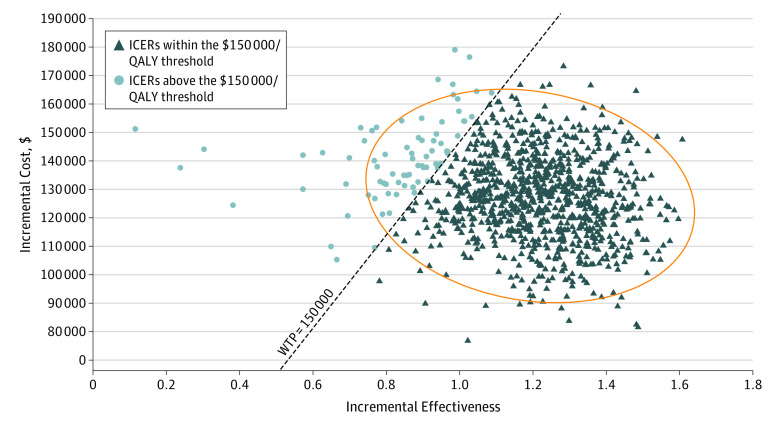
Cost-Effectiveness With Probabilistic Sensitivity Analysis Incremental cost-effectiveness ratios (ICERs) from 10 000 simulations in probabilistic sensitivity analysis. Quality-adjusted life-year (QALY) threshold indicated by dashed line. In all simulations, adjuvant olaparib was both more effective and more costly than no adjuvant olaparib. The oval depicts a 95% confidence ellipse. WTP indicates willingness to pay.

**Figure 3.**
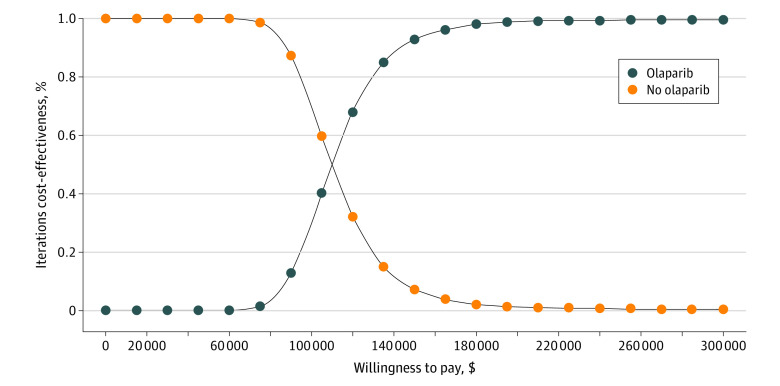
Cost-Effectiveness Acceptability Curve Curves indicate the proportion of simulations (n = 10 000) in probabilistic sensitivity analysis for which each strategy is preferred, as a function of the threshold willingness to pay for an additional quality-adjusted life-year.

In probabilistic sensitivity analysis, we found that, in all 10 000 simulations, adjuvant olaparib was both more costly and more effective than no olaparib (Figure 2). In more than 92% of the simulations, olaparib was preferred to no olaparib at a willingness-to-pay threshold of $150 000 per QALY gained. The probability that adjuvant olaparib was cost-effective, compared with no olaparib, exceeded 50% at a willingness-to-pay threshold of approximately $111 000 per QALY and exceeded 80% at a threshold of approximately $130 000 per QALY (Figure 3). At a threshold of $100 000 per QALY, the probability of adjuvant olaparib being cost-effective was approximately 30%.

## Discussion

Since the approval of trastuzumab for *ERBB2*-positive tumors in 1998, targeted systemic therapy remains a paramount goal of drug discovery and clinical care for breast cancer treatment.^20^ PARP inhibitors represent one of the newer classes of targeted therapies for breast and other solid tumors. The OlympiA trial indicates that adjuvant olaparib improves both DDFS and OS in patients with high-risk, early-stage breast cancer with gBRCAm. Our economic evaluation suggests that olaparib is also cost-effective in this setting.

In our base-case analysis from a health care system perspective, adjuvant olaparib was associated with an ICER of approximately $111 000 per QALY gained. This estimate compares favorably with currently accepted thresholds of willingness to pay for health gains in the US and with estimates of the cost-effectiveness of olaparib in other tumor types and settings.^21^ In its original indication for recurrent ovarian cancer in women with gBRCAm, estimates of the cost-effectiveness of olaparib in the US have varied from approximately $193 000 to $287 000 per progression-free year of life saved.^22,23,24^ For newly diagnosed advanced ovarian cancer, the ICER could be as low as $51 000 per QALY.^25^ In metastatic prostate cancer, the estimated cost-effectiveness of olaparib has varied from approximately $117 000 to just under $250 000 per QALY.^26,27^

It is notable that more than 80% of patients in each arm of the OlympiA trial had TNBC. Given the relatively poor prognosis for patients with this tumor profile, adjuvant therapies that can reduce recurrence risk in TNBC are especially meaningful. Not only did olaparib have clinical benefit in this patient population, our results suggest that, despite its relatively high cost, it also represents a good value.

Our results were sensitive to assumptions about the effectiveness of olaparib. Within the 95% CI of the HR for DDFS found in the OlympiA trial, the ICER for adjuvant olaparib varied nearly 3-fold, from as low as $76 000 per QALY gained to as high as $212 000 per QALY gained. Now that olaparib is approved for adjuvant breast cancer treatment in patients with gBRCAm, it will be important to monitor its effectiveness and cost-effectiveness in the clinical practice setting.^1^

Our model was agnostic to specific therapies received in the metastatic setting. We used an average monthly cost estimate for treating metastatic TNBC based on a mix of agents and regimens, which may have included a PARP inhibitor, and our estimate of breast cancer mortality following distant recurrence was based on evidence from cohorts with TNBC who were treated before the advent of PARP inhibitors. It is not yet clear whether oncologists will routinely administer PARP inhibitors in the metastatic setting following disease recurrence in patients who received adjuvant olaparib. As physicians begin incorporating adjuvant olaparib into their treatment protocols, we will learn more about the subsequent use and effectiveness of PARP inhibitors for treating metastatic disease. It is also not clear how the effectiveness and cost-effectiveness of adjuvant olaparib might be affected by the use of other agents, including pembrolizumab, capecitabine, and abemaciclib. Some of these medications have become standard of care in the neoadjuvant or adjuvant settings for patients with TNBC or other tumor features.^28^ As newer data on costs and outcomes of different treatment combinations and sequences become available, estimates of olaparib effectiveness and cost-effectiveness can be updated to consider the impact of these other systemic therapies.

### Limitations

This study has several limitations. We did not explicitly model local and regional recurrences or subsequent primary cancers. Although these events were uncommon during the available follow-up, and the OlympiA trial was not powered to test differences in these end points, observed rates favored the adjuvant olaparib arm. Similarly, we did not explicitly model adverse events or treatment discontinuation. The rate of serious adverse events—events causing death, hospitalization, or major disability—was similar in the 2 study arms. While the frequency of adverse events leading to treatment discontinuation was greater among patients receiving olaparib (10.8% vs 4.6% of those receiving placebo), the absolute difference was relatively small. Given the substantial impact of olaparib on DDFS and OS, explicitly incorporating treatment discontinuation would not likely alter our conclusions. Moreover, our base-case analysis assumed that adjuvant olaparib was, on average, associated with slightly diminished quality of life compared with no olaparib. Although there was no significant difference between treatment arms in quality of life during the first 24 months following randomization in the OlympiA trial, more patients in the olaparib arm experienced a grade 3 or 4 adverse event, particularly anemia. Even when the utility weight for olaparib was assumed to be as low as 80% of the utility of no olaparib (ie, olaparib utility, 0.784; no olaparib utility, 0.98), the ICER for adjuvant olaparib was less than $150 000 per QALY gained.

In some instances, we used Medicare reimbursement rates as a proxy for health care costs, although most patients in the model would not be eligible for Medicare, since we assumed a starting age of 42 years, consistent with the OlympiA trial.^2^ However, sensitivity analysis on these model inputs—costs of routine oncology visits and surveillance mammograms and breast magnetic resonance imaging—suggested that they had almost no impact on the estimated ICER of adjuvant olaparib.

In extrapolating results beyond the OlympiA trial, we conservatively assumed that olaparib had no association with DDFS and OS after 4 years. If olaparib has longer-term benefit, then our analysis would yield an overestimate of the ICER, and therefore an underestimate of the cost-effectiveness of the drug in this setting.

In addition, our base-case analysis was conducted from the health care system perspective, and thus did not include costs associated with informal caregiving or lost productivity. Patients receiving olaparib might incur greater costs associated with lost workdays due to a greater number of physician visits during the year of treatment. However, these costs would be insubstantial compared with the costs of informal caregiving in patients at the end of life and productivity losses associated with morbidity and premature mortality due to metastatic cancer. Such productivity losses could be considerable given the age distribution of these patients with breast cancer.^29^ However, since adjuvant olaparib should decrease these costs on average by reducing the risk of metastatic recurrence, an analysis conducted from a societal perspective would likely find a more favorable ICER.

We assumed that gBRCAm status is known for every patient entering the model. Despite calls for universal germline genetic testing in all patients with newly diagnosed breast cancer, National Comprehensive Cancer Network guidelines still recommend testing based on risk factors associated with clinical presentation, personal health history, and family history.^30,31,32^ The availability and effectiveness of adjuvant olaparib for patients with gBRCAm is likely to prompt increased testing among patients with newly diagnosed early-stage breast cancer. A recent study of strategies to identify patients with breast cancer eligible for olaparib treatment reported that universal testing in patients with TNBC was cost-effective (ICER<$60 000 per QALY) compared with a selective testing strategy, and expanding testing to all patients with ERBB2-negative disease had a favorable ICER as well.^33^ Universal germline testing might have even greater value when cascade testing in family members is considered. However, ensuring equitable access to adjuvant olaparib may require dedicated efforts to reduce racial disparities in the use of germline genetic testing.^5,34,35^

## Conclusions

Expensive new cancer drugs such as olaparib can pose a challenge for both payers and patients.^36,37^ Ensuring access to this effective and cost-effective agent is critical if we are to attenuate, rather than exacerbate, disparities in breast cancer outcomes. This is especially important in the context of TNBC, which is disproportionately common and lethal in Black women.^38,39^ The findings of this economic evaluation suggest that with an ICER less than $150 000 per QALY gained, adjuvant olaparib in eligible patients represents a good value at its 2021 price.

## Notes

Supplement.
Data Sharing Statement
