# Supplementary material for: Cost-Effectiveness of Adjuvant Olaparib for Patients With Breast Cancer and Germline BRCA1/2 Mutations
Source: JAMA Netw Open. 2024 Jan 3;7(1):e2350067. doi: 10.1001/jamanetworkopen.2023.50067 (PMC10765260; doi:10.1001/jamanetworkopen.2023.50067)
Supplement: Supplement. — Data Sharing Statement [file jamanetwopen-e2350067-s001.pdf]

Data Sharing Statement

Zettler. Cost-Effectiveness of Adjuvant Olaparib for Patients With Breast Cancer and Germline BRCA1/2 Mutations. *JAMA Netw Open*. Published January 03, 2024.
doi:10.1001/jamanetworkopen.2023.50067

Data
Data available: Yes
Data types: Other (please specify)
Additional Information: The datasets generated or analyzed during the current study are available from the corresponding author on reasonable request.
How to access data: elena.elkin@columbia.edu
When available: With publication

Supporting Documents
Document types: None

Additional Information
Who can access the data: Researchers requesting it for non-commercial purposes.
Types of analyses: Non-commercial purposes.
Mechanisms of data availability: After approval by the corresponding author.
